# Neurodegenerative Disorder Risk in Krabbe Disease Carriers

**DOI:** 10.3390/ijms232113537

**Published:** 2022-11-04

**Authors:** Lorenza Vantaggiato, Enxhi Shaba, Alfonso Carleo, Daiana Bezzini, Giovanna Pannuzzo, Alice Luddi, Paola Piomboni, Luca Bini, Laura Bianchi

**Affiliations:** 1 Functional Proteomics Laboratory, Department of Life Sciences, University of Siena, 53100 Siena, Italy; 2 Department of Pulmonology, Hannover Medical School, Carl-Neuberg-Straße 1, 30625 Hannover, Germany; 3 Department of Life Sciences, University of Siena, 53100 Siena, Italy; 4 Department of Biochemical and Biotechnological Sciences, Section of Physiology, University of Catania, 95121 Catania, Italy; 5 Department of Molecular and Developmental Medicine, University of Siena, 53100 Siena, Italy

**Keywords:** globoid cell leukodystrophy, neurodegeneration, neuroinflammation, demyelination, ubiquitin dependent degradation, multiple sclerosis, Alzheimer disease, Parkinson disease, Lewy body

## Abstract

Krabbe disease (KD) is a rare autosomal recessive disorder caused by mutations in the galactocerebrosidase gene (*GALC*). Defective GALC causes aberrant metabolism of galactolipids present almost exclusively in myelin, with consequent demyelinization and neurodegeneration of the central and peripheral nervous system (NS). KD shares some similar features with other neuropathies and heterozygous carriers of *GALC* mutations are emerging with an increased risk in developing NS disorders. In this work, we set out to identify possible variations in the proteomic profile of KD-carrier brain to identify altered pathways that may imbalance its homeostasis and that may be associated with neurological disorders. The differential analysis performed on whole brains from 33-day-old twitcher (*galc ^−/−^*), heterozygous (*galc ^+/−^),* and wild-type mice highlighted the dysregulation of several multifunctional factors in both heterozygous and twitcher mice. Notably, the KD-carrier mouse, despite its normal phenotype, presents the deregulation of vimentin, receptor of activated protein C kinase 1 (RACK1), myelin basic protein (MBP), 2′,3′-cyclic-nucleotide 3′-phosphodiesterase (CNP), transitional endoplasmic reticulum ATPase (VCP), and N-myc downstream regulated gene 1 protein (NDRG1) as well as changes in the ubiquitinated-protein pattern. Our findings suggest the carrier may be affected by dysfunctions classically associated with neurodegeneration: (i) alteration of (mechano) signaling and intracellular trafficking, (ii) a generalized affection of proteostasis and lipid metabolism, with possible defects in myelin composition and turnover, and (iii) mitochondrion and energy supply dysfunctions.

## 1. Introduction

Krabbe disease (KD) (OMIM #245200), also known as globoid cell leukodystrophy, is a neurological disorder presenting demyelination and impaired neuronal transmission. It is a rare autosomal recessive disease that affects the central and peripheral nervous system (CNS and PNS, respectively). Infantile, juvenile, and adult-onset forms of KD have been described, with the infantile form being the most common, rapidly progressive, and fatal while the latter presenting less severity and slower progression [1,2].

KD is a lysosome storage disease (LSD) caused by the functional deficiency of galactocerebrosidase (GALC). This lysosomal enzyme is vital for the degradation of sphingolipids, for the recycling of sphingosine and ceramide, and, consequently, for myelin synthesis and homeostasis [3,4,5,6]. Biochemical defects related to GALC aberrant functionality cause abnormal myelin turnover that leads to demyelination, neurodegeneration, and neuroinflammation, with white matter perivascular monocyte/macrophage infiltrates [7].

Although it is not unique in catabolizing the myelin highly-abundant sphingolipid galactosylceramide (GalCer), GALC is the only known enzyme able to degrade galactosylsphingosine (psychosine, Psy), whose accumulation is highly toxic [8]. Nonetheless, increasing evidence suggests that all the dysfunctions described in KD cannot be merely explained by psychosine accumulation and that additional mechanisms could be involved. In fibroblasts from KD patients, Papini et al. [9] have recently correlated increased concentrations of lactosylceramide (LacCer), a bioactive lipid metabolized by GALC, to several signaling pathway alterations involved in neurodegeneration. Other studies have also reported axonal defects independent of Psy accumulation or demyelinization [10,11]. In fact, Kreher et al. [11] proved that GALC absence in neurons triggers neuro-axonal degeneration and related inflammatory demyelination. In addition, neuron-specific GALC ablation was described in attenuating neuron maturation during brain development [12], and thus intimately impacting the CNS properties.

Phenotypically, KD shares similar traits with other neurodegenerative disorders that, in some cases, correlate to similar molecular defects. Notably, several papers report KD patients, prevalently with late onset KD, and misdiagnosed as affected by MS. Sahai et al. [13] discussed the MS family history of a KD patient with infantile-onset KD. In addition, genome-wide association studies (GWAS) and expression analyses recognized, in the *GALC* SNPs and expression levels, highly significant patterns distinguishing a number of people with MS (PwMS) from healthy controls [14,15,16]. Currently, the *GALC* gene (14q31) is actually considered a susceptibility locus for MS [15]. Furthermore, α-synuclein fibrillization and Lewy body (LB) formation, which are characteristic features of synucleinopathies, lipidoses, other LSDs, and mitochondrial diseases, were described in KD human and mouse brains [17,18].

Despite the very low incidence of KD [1:100,000 live births in the United States and Europe, with a pick of incidence recorded in Sweden (1:39,000)] [19,20], heterozygous carriers are estimated to be frequent in Caucasians (1:150) [21] and are suggested to carry a risk for neurological disorder onset [5]. In fact, despite carriers of recessive disorders not being clinically ill, they may present non-physiological biochemical and molecular traits that may predispose them to develop a disease state [16]. In fact, carriers of mutations causing KD are known for presenting an increased risk for developing open angle glaucoma and pulmonary artery enlargement in chronic obstructive pulmonary disease [22,23]. Furthermore, the *galc ^+/−^* mutation carrier mouse, from the twitcher (Twi) KD-model, seems to corroborate these hypotheses. After cuprizone administration, young adult heterozygous mice, despite being apparently healthy and identical, in physiological conditions, to wild-type (WT), present a reduced remyelination response and an impaired microglial reaction to myelin damage when compared with similarly treated WT animals. These defects result in a reduced debris clearance and a more frequent inflammation occurrence, which may increase the possibility to develop auto-antibodies against components of myelin sheaths [16]. In addition, a late onset manifestation of GALC deficiency in Twi mice underwent massive gene therapy (*galc* transgene) consisting of brain lesions associated with leakage of the blood–brain barrier and plasma fibrinogen extravasation (PFE), with consequent activation of the fibrinogen-bone morphogenic protein (BMP)-SMAD-glial fibrillary acidic protein (GFAP) glia response [24]. In particular, PFE observed in late Twi treated-mice, where *galc* transgene decreased in expression, resembles microglial activation and brain damage observed in neurological disorders. Asymptomatic *galc ^+/−^* heterozygous mice also present, similarly to Twi animals, an increase in oxidative stress markers, i.e., F2-isoprostanes and F4-neuroprostanes, that were generated from polyunsaturated fatty acid oxidative damage. Their occurrence is retained impacting on the nervous system (NS) and associated with neurological diseases [25].

Based on these premises, we performed a functional proteomic analysis on whole brain lysates from 33-day-old Twi, heterozygous *galc* ^+/−^ carrier (Het), and wild-type (WT) mice [26] to identify molecular affections and related biomarkers that, over time, may increase or report an increased risk for carriers to develop NS affections. As proteoform heterogeneity define protein properties and the functional profile of investigated systems, we applied a 2DE/mass spectrometry approach with the intent to capture and visualize, on a large scale, possible different proteoform presences that may differentially impact mutants’ physiology.

According to our data, the Het proteome profile diverges from both WT and Twi ones. Hence, we provide an integrated overview on the identified protein differences to delineate their eventual correlation to biochemical and molecular aberrances previously described in other neuropathologies. Our data suggest significant dysfunctions in different pathways, critical for CNS homeostasis, occurring in Het brain. These are likely to make KD carriers more vulnerable to any other risk factor, either environmental or genetic, thus exposing them to a greater likelihood of developing neuropathies.

## 2. Results and Discussion

**Figure 1:**
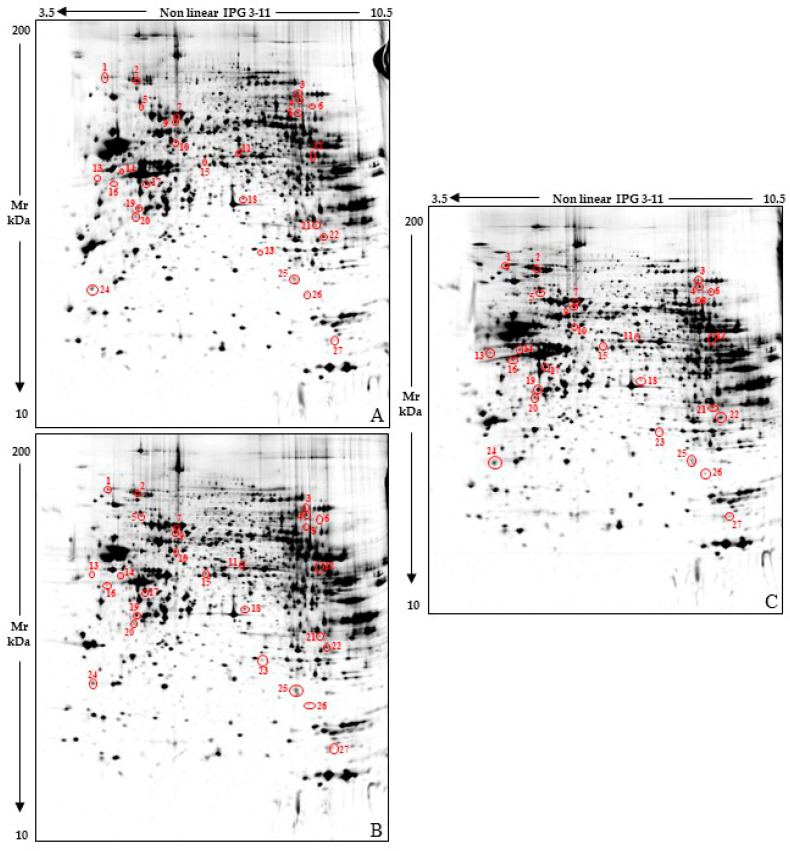
Reference whole brain lysate protein patterns of 33-day-old wild-type (WT) (**A**), heterozygous *galc ^+/−^* (Het) (**B**) and Twitcher *galc ^−/−^* (Twi) (**C**) mice. Red circles and numbers point out differentially abundant protein spots detected among the three sample classes. Numbers matched those listed in Table 1 and Figure 2.

**Figure 2:**
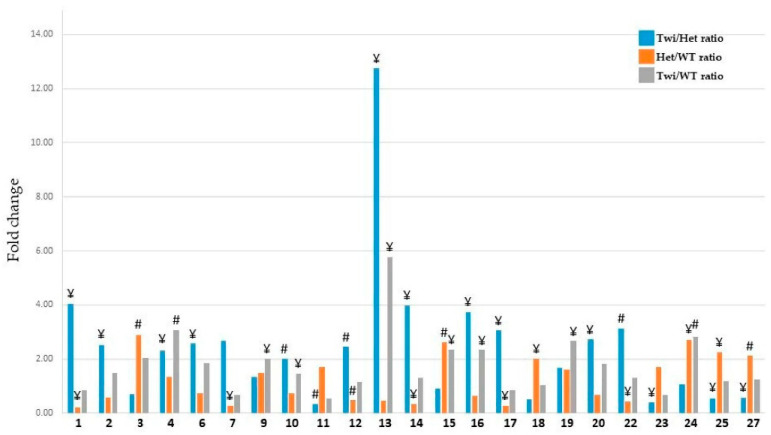
Histogram representing the %Vol ratios of Twi vs. Het (blue bar), Het vs. WT (orange bar), and Twi vs. WT (grey bar) as reported in Table 1. The numbers in the x axis correspond to those of differentially abundant protein spots identified by mass spectrometry, for more clarity: 1: Ubiquitin carboxyl-terminal hydrolase 5; 2: Transitional endoplasmic reticulum ATPase; 3: Aconitate hydratase, mitochondrial; 4: Aconitate hydratase, mitochondrial; 6: Aconitate hydratase, mitochondrial; 7: Albumin; 9: Dihydrolipoyllysine-residue acetyltransferase component of pyruvate dehydrogenase complex, mitochondrial; 10: Dihydropyrimidinase-related protein 2; 11: Lipoamide acyltransferase component of branched-chain alpha-keto acid dehydrogenase complex, mitochondrial; 12: 2′,3′-cyclic-nucleotide 3′-phosphodiesterase; 13: Vimentin; 14: ATP synthase subunit beta, mitochondrial; 15: Protein NDRG1; 16: NSFL1 cofactor p47; 17: Tropomodulin-2; 18: 60S acidic ribosomal protein P0; 19: Beta-soluble NSF attachment protein; 20: Creatine kinase B-type; 22: Receptor of activated protein C kinase 1; 23: Protein-L-isoaspartate(D-aspartate) O-methyltransferase; 24: Translationally-controlled tumor protein; 25: Transgelin-3; 27: Myelin basic protein. # and ¥ indicate statistical relevance, i.e. *p* ≤ 0.01 and 0.01 < *p* ≤ 0.05, respectively.

We applied a functional proteomic approach on whole brain protein extracts obtained from 33-day-old twitcher, *galc* ^+/−^ heterozygous, and wild-type mice. At this age, the disease is overt in Twi and GALC activity drastically lower than in the controls [27]. Nonetheless, they are still viable since death occurs by ~40 days after birth. Twenty-seven highly significant protein differences were detected among these sample classes (Figure 1) by applying 2DE and gel image analysis (on seven gels for each tested condition), rigorous statistics, and result filtering for %Vol fold change. Then, MALDI-TOF/TOF mass spectrometry successfully identified 23 of the differing protein spots, which correspond to 21 unique proteins (Table 1 and Figure 2).

**Figure 3:**
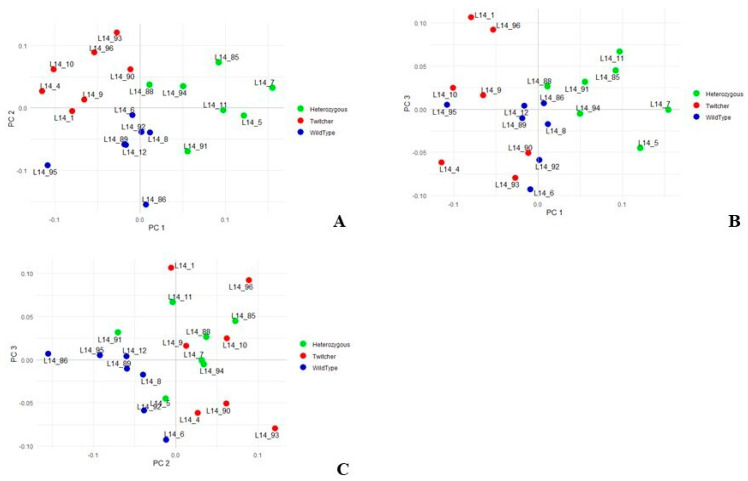
Principal component analysis (PCA) performed on %Vols of spots matched among WT, Het, and Twi mouse samples. Plots highlight spatial distribution of the 21 whole brain analyzed samples—7 from WT mice (blue symbols); 7 from Het mice (green symbols); and 7 from Twi mice (red symbol)—along the PC1 and PC2 (**A**), PC1 and PC3 (**B**), and PC2 and PC3 (**C**).

The variance-covariance analysis was performed by principal component analysis (PCA) on the %Vol values of significantly differing spots and the first three axes of variation, i.e., PC1, PC2, and PC3, explain the 27.5%, 19.8%, and 13.1% of variance, respectively. The PC1/PC2 plot (Figure 3) shows Het mice clustering apart from the others on the right side of PC1 and partially overlapping with the Twi and WT groups around the center of PC2 (Figure 3A). The WT cluster is instead interposed, on PC1/PC2 (Figure 3A) and PC1/PC3 (Figure 3B) plots, between the other two conditions and presents a relatively low PC1 variability with a relevant clustering. Finally, Twi and Het mice widely overlap on the PC1/PC2 and partially on PC2/PC3 plots throughout PC2 and PC3 axes, respectively (Figure 3C).

Overall, PCA evidences that Het animals, despite being apparently “normal”, present a distinct biochemical profile from Twi and WT mice, which may expose Het to an increased risk to develop other disorders, including neuropathologies [28].

To further the understanding of the biological implications that the protein profile outlined in Het may have, we performed a bioinformatics analysis and predicted functional interactions occurring between the 21 differentially identified abundant unique proteins by applying the shortest-path-network (SPN) building tool from the MetaCore resource. Except for tropomodulin-2, all the processed proteins entered into the SPN, resulting in being perfectly embedded in their functionality. This underlines that identified factors belong to the same or related biochemical pathways.

**Figure 4:**
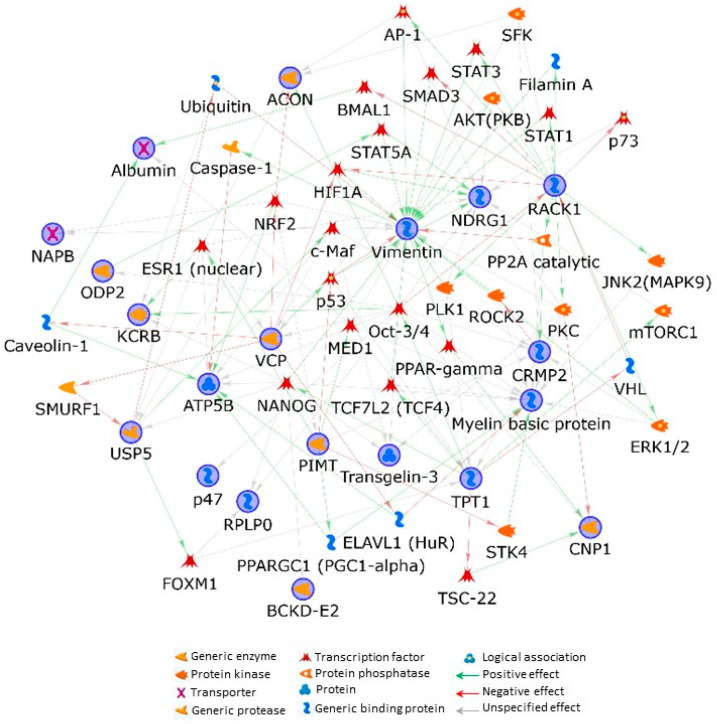
MetaCore protein network built by processing significant protein differences occurring among Twi, Het, and wild-type mice. Experimental proteins, circled in blue, were cross-linked by using the shortest-path-network (SPN) building tool. This generates hypothetical networks by cross-linking experimental factors and expanding protein interactions to other proteins, not present in the processed experimental list but supported by the MetaCore database, that are needed to functionally correlate user up-loaded proteins, which do not directly interact. Except for tropomodulin-2, all the processed proteins entered into the SPN.

Vimentin (VIME), protein NDRG1 (alternative name: N-myc downstream-regulated gene 1 protein), receptor of activated protein C kinase 1 (RACK1), transitional endoplasmic reticulum ATPase (alternative name: valosin-containing protein, VCP), and myelin basic protein (MBP) emerged as central hubs, by establishing the highest number of interactions with the other proteins of the net (Figure 4). Furthermore, the MetaCore enrichment analysis in the “Pathways Maps” ontology, which highlights the molecular pathways related to identified differences (Table S1), suggested the presence of cytoskeleton remodeling and neuronal cell development affections and their direct correlation to VIME and MBP deregulation in Twi as well as in Het mice.

### 2.1. Cytoskeleton (Re)Organization and Its Possible Roles in Myelination Dynamics of KD Carriers

**Figure 5:**
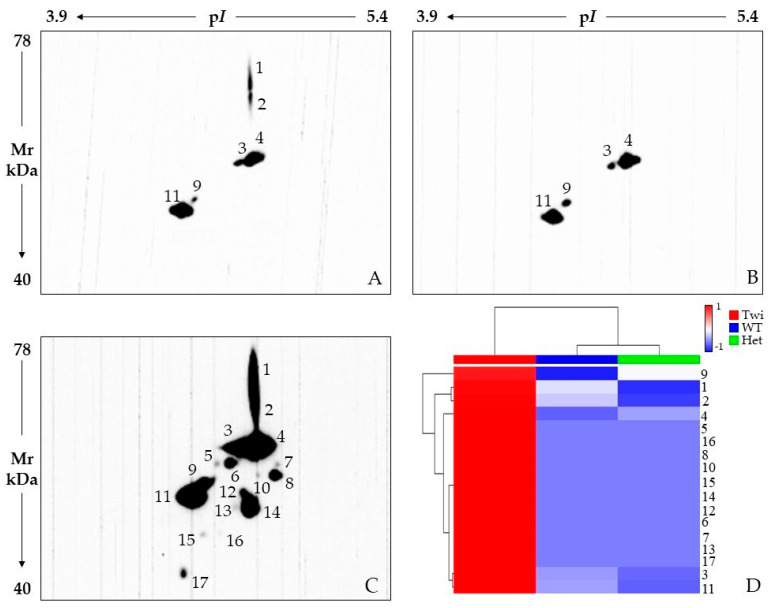
2D Western blot analysis with anti-vimentin antibody on 33-day-old wild-type (WT) (**A**), heterozygous *galc* ^+/−^ (Het) (**B**) and twitcher *galc* ^−/−^ (Twi) (**C**) mice. Heatmap analysis (**D**) scaled values of spot intensity. Color changes from blue to red indicate less or higher signal intensity, respectively. Each row corresponds to a VIME protein species while each column corresponds to one of the three tested conditions, as highlighted by the colored bar from the horizontal dendrogram: Twi = red, WT = blue, and Het = green. Row spot numbers match those in (**A**–**C**).

Vimentin spot 13 is the most differing protein spot among the significant differences we detected (Table 1, Figure 2), by showing fold changes of 12.75 and 5.77 in the Twi vs. Het and Twi vs. WT comparisons, respectively. In addition, the anti-VIME 2D WB, shown in Figure 5, evidenced the presence of several VIME spots exclusive to the Twi mouse. A complex VIME pattern, not merely limited to the protein abundance of spot 13, evidently distinguishes the Twi from Het and WT animals. This type III intermediate filament (IF) is an astrocyte marker whose increased expression characterizes reactive astrocytosis and that was previously described in CNS and PNS of 30/33-day-old Twi mice [29]. Although the effective role of its increase has not been defined, vimentin was proved to participate in CNS damage-restraint by sequestering affected areas [30]. Interestingly, its down-regulation was also reported as having pathological effects by negatively regulating myelination [31] and causing several astrocyte dysfunctions during cell migration, vesicle trafficking, oxidative stress response, reconstruction of the blood–brain barrier, and interaction with microglia [31]. On the one hand, the very high up-regulation of vimentin in Twi can derive from an extensive astrocytosis following wide myelin damage. On the other hand, the decrease in the spot 13 proteoform (Figure 1), although not significant, the lack of the immunosignals 1 and 2 (Figure 5A–C), and the general intensity decrease in various immunosignals (Figure 5A–C) in Het animals may imply the occurrence of differences in VIME processing and in related cytoskeleton structure and dynamics between the WT and Het mice. The immunosignal intensity heatmap (Figure 5D) and the multiple line chart (Supplementary Figure S1) visualize an overall affection of the vimentin pattern in Het, which differs from both WT and Twi mice.

Cytoskeleton defects are known to impair numerous cell functions, such as mechanosignaling, migration, differentiation, proliferation, intracellular trafficking, and mitochondria metabolism and activities. Several of these processes have been described as defective in KD [31,32,33]. In particular, abnormal cytoskeleton is widely associated to neurodegeneration [34,35] and aberrances in VIME post-translational modifications, e.g., phosphorylation, have been described in some degenerative neuropathies, such as Niemann–Pick disease Type C1 [36] and Huntington’s disease [37], or as a marker of activated astrocytes, i.e., citrullination, in Creutzfeldt–Jakob disease [38].

The relevance of this IF in the pathophysiology of KD advanced stage and maybe in affections of young adult carriers is further stressed by the MetaCore SPN, where VIME resulted in the main central hub. SPN also evidences a tight indirect regulation of VIME by RACK1, another main hub of the net. RACK1 is actually suggested modulating VIME by controlling STAT1, STAT3, AP-1, HIF1A, and SMAD3 transcription factors, PKC and AKT kinases as well as the protein phosphatase 2A (PP2A catalytic), and the actin-network component filamin-A. Notably, all these interactors are known to play key roles in NS physiology and their dysfunctions have been associated with several neuropathies, including tauopathies [39,40,41,42,43,44,45,46,47,48,49].

Besides its functional correlation with VIME, RACK1 acts as a neuroprotective factor [50,51] whose down-regulation (Table 1, Figure 2, spot 22) further stresses the possible occurrence of myelin dyshomeostasis related to cytoskeleton dysfunctions in Het animals. As a plectin-binding protein, RACK1 is in fact thought linking constituents of different signaling pathways to the cytoskeleton and mediating signal transmission to cytoskeleton remodeling machineries [52]. Among others, the above mentioned PKC is anchored to the cytoskeleton and held in active conformation by interacting with this scaffold protein [53]. Interestingly, dysfunctions in PKC anchoring caused by defects in RACK1 have been associated to aging and to AD pathophysiology [52]. After all, RACK1 dependent PKC signal transduction regulates the MBP phosphorylation [54,55] whose defects can cause demyelination in MS [56]. The RACK1 down-regulation detected in Het animals may hence represent a potential molecular link between the heterozygote *GALC* mutant and the risk for developing MS in humans, principally related to the failure of remyelination in progressive MS [5,14,57,58].

MBP is a highly abundant and essential membrane stacker in CNS and PNS myelin [59]. Smirnova et al. [60] have recently delineated the wide interatomic pattern of MBP, which reveals an incredible, heterogeneous, and complex plethora of functions in which this intrinsically disordered protein may act. Among others, the MBP-interacting net includes cytoskeleton components and proteins involved in the reorganization of the actin cytoskeleton at the plasma membrane and cell adhesions [60]. By linking the underling cytoskeleton and proteins containing SH3 domains to the membrane, it is also active in transmembrane signal transduction to the cytosol and, consequently, in myelinating cell response control [61].

MBP molecular interactors and related functions are intimately defined by the maturation processes it undergoes. MBP presents a complex proteoform pattern, resulting from alternative splicing and differential post-translational modifications, that profoundly influences myelin physiology and related pathology. Besides the above mentioned phosphorylation, MBP citrullination levels correlate with myelin sheath instability and MS pathogenesis and severity [62,63,64].

The abundance of the MBP-proteoform spot difference we identified (Table 1, Figure 2, spot 27) is considerably increased in Het with respect to both Twi and WT animals (Table 1, Figure 2). Since this structural myelin protein is the only known factor indispensable to generate compact myelin sheaths in the CNS and in reason of its aforementioned functional plasticity related to its post translational modifications (PTMs), the MBP proteoform observed increase in KD carriers could have a biochemical echo in myelin stability and integrity maintenance [65].

On the same line of reasoning, a probable axon dysfunction and dyshomeostasis process in myelinating cells from Het mice may also be supposed in relation to the significant decrease in 2′,3′-cyclic-nucleotide 3′-phosphodiesterase (CNP) that we detected occurring between Het and WT animals (Table 1, Figure 2, spot 12). This protein is an oligodendrocyte early differentiation marker with microtubule-associated-protein (MAP) like functions that was reported as counteracting MBP by organizing the F-actin cytoskeleton to prevent cytosolic membrane leaflet collapsing in the multilayered myelin [66]. Although its function is not fully understood, CNP is thought to play a crucial role in myelin physiology [67]. It is in fact essential for murine axonal survival but not for myelin assembly, and adult CNPase-null mice present a normal myelin morphology but develop neurodegeneration [68]. Interestingly, Al-Abdi et al. [66] reported a normal development before hypomyelinating leukodystrophy and premature death in consanguineous patients presenting CNP deficiency. In addition, erythrocytes from PwMS have lower CNP activity than control cells [69]. In PwMS, this protein was also recognized as a target of self-antigens and described as involved in the pathological demyelination of axon terminals [70].

Our hypothesis on a probable cytoskeletal rearrangement, with reasonable neurological effects, in carrier brains is further strengthened by the detected deregulation of other cytoskeletal modulators, i.e., NDRG1, the translationally-controlled tumor protein (TCTP), tropomodulin-2, and transegelin-3 (Table 1, Figure 2).

The latter (spot 25), up-regulated in Het mice compared to both Twi and WT animals, is an actin and tubulin co-localizing protein of the NS that is considered relevant in neuron plasticity [71] and in maintaining neuronal morphology [72], which was confirmed to be down-regulated in the brain of Alzheimer’s disease (AD) patients [73].

In addition, tropomodulin-2, decreased in Het mice (spot 17), is a neuron-specific regulatory protein that prevents elongation and depolymerization of actin filaments [74].

NDRG1 (spot 15) and TCTP (spot 24), significantly up-regulated in Het and Twi mice when compared to WT animals, control instead microtubule dynamics [75,76,77]. The SPN central hub NDRG1, conceivably by regulating also lipid metabolism [78], is crucial for the development and maintenance of Schwann myelin sheath and of adult myelinating oligodendrocytes. Its deficiency leads to Schwann cell dysfunction and may result in greater susceptibility of myelin to damage in PwMS brain [79,80]. Noteworthy, the SNP evidenced an indirect functional correlation existing between RACK1 and NDRG1. Specifically, the former controls the latter through the serine/threonine-protein kinase PLK1 (PLK1), AKT/PKB, PKC, and the transcription factors p73, AP-1, and HIF1A. As stated above, several of these factors, as well as other SPN transcription factors (i.e., the estrogen receptor ESR1, p53, and STAT5A) functionally linked to NDRG1 but not under the direct RACK1 activity (Figure 4), have been reported to be involved in KD or in other neurodegenerative disorders [78,81,82,83,84,85,86,87,88,89,90,91,92,93,94]. Not coincidentally, NDRG1 expression and activity may depend on different endogenous and environmental signals, such as cell differentiation, p53-mediated apoptosis, hypoxia, and endoplasmic reticulum (ER) stress [95,96,97,98,99,100], which are aberrant or increased in KD patients and that have been correlated to psychosine accumulation [25]. Moreover, NDRG1 is widely described as dysregulated in Charcot–Marie–Tooth disease type 4, which presents severe and progressive demyelination, and, with other NDRG family members (e.g., NDRG2), it might influence other neurodegenerative disorders such as AD, the above mentioned MS, and frontotemporal lobar degeneration [78,101]. This evidence may be relevant in evaluating the risk for KD carriers to develop neuropathies related to defective cellular processes involving NDRG1 functions in ER stress response, cell trafficking, and lipid metabolism, as further discussed below [78,102,103].

### 2.2. Lipid Metabolism Defects in Krabbe Disease

As already stated, KD is an inborn error of lipid metabolism. Consequently, the factors involved in lipid metabolism that we have identified as differing between mutants and WT mice may help to clarify how the defective metabolization of GALC substrates radiates to other lipid metabolism processes, and definitely compromises NS lipid homeostasis.

Sphingolipids, in particular sulfatides and their precursor galactosylceramide (GalCer), are one of the main representative components of myelin. Studies in mice reported GalCer as an essential factor for neural development, for the maintenance of myelin stability, and having protective functions in both CNS and PNS [3]. GalCer is highly abundant in compact myelin and, despite myelinating cells being able to compensate for defects in lipid synthesis, GalCer and sulfatide defective synthesis causes a failure of the long-term stability of myelin [4].

Furthermore, the role of sphingolipids are not limited to structural functions: they have been reported modulating signal transduction, differentiation, apoptosis, autophagy, and necrosis [48]. GalCer, in particular, increases the thickness of raft membranes and affects membrane viscosity between its two leaflets thus interfering in membrane protein composition and activation, in membrane interactions with cytoplasmic structural proteins, and in signal transduction [6]. Sphingolipids may also be metabolized in signaling molecules that may regulate the immune and nervous systems and whose receptor targeting has been applied to treat MS [104].

Evidently, sphingolipid metabolism defects may profoundly impact myelin composition, organization, stability, and functionality [5]. In this context as well as the MBP up-regulation discussed above, it may be related to myelin disorganization in association with the altered lipid composition of myelin [74].

Moreover, Psy accumulation in lipid raft disrupts their organization, increases their cholesterol concentration, and modifies the distribution of two protein markers of these domains: flotillin-2 and caveolin-1 [105,106,107]. The latter was also provided by SPN (Figure 4) as directly linked with the mitochondrial ATP synthase subunit beta (ATPB), a protein that we detected to be down-regulated in Het mice when compared to both Twi and WT animals (Table 1, Figure 2, spot 14). Interestingly, ATPB is involved in lipoprotein uptake [108] and its expression was observed as increasing in Alzheimer’s and Huntington’s diseases [109].

Among the proteins deregulated in Het mice, numerous evidences describe the multi-functional NDRG1 also involved in lipid homeostasis by acting as an adipogenesis promoter [110], and as an important factor for oligodendrocyte cholesterol balance and differentiation [111,112]. Interestingly Pietiäinen et al. [112] also reported a significant increase in the total ceramide levels in NDRG1 depleted cells. In light of these findings and in particular for its correlation to ceramide, NDRG1 deregulation in Het and Twi animals supports the hypothesis of lipid metabolism defects and associated brain dysfunctions also in Het mice.

Recently, attention has turned to the role that dihydropyrimidinase-related protein 2 (DPYSL2), another protein up-regulated in Twi animals (Table 1, Figure 2, spot 10), seems to play during adipogenesis and lipid metabolism in metabolic disorders related to neurodegeneration. It is typically expressed in adult neurons and oligodendrocytes and represents the major phosphoprotein in neuronal tissues where it acts in microtubule stability, neuronal outgrowth, cellular polarity, and vesicle trafficking [113]. Chang et al. [114] proved DPYSL2 correlation to energy metabolism in adipogenesis and lipid deposits and to cytoskeleton remodeling necessary for lipid maturation, hence suggesting a molecular link between neurodegenerative diseases and metabolic disorders. Of relevance, DPYSL2 modulation by molecular therapy has been reported to improve symptoms of AD in animal models [115]. DPYSL2 may indeed represent an interesting molecular checkpoint for novel pharmacological attempts, especially in late-onset KD.

### 2.3. Proteostasis Affection in Het Mice

The proteostasis network (PN) results from a fine balance of synthesis, folding, PTM, quality control, localization, and degradation of proteins and it is fundamental for cellular physiology [116]. ER plays a pivotal role in protein homeostasis and has emerged as a key compartment for brain development and function [117]. Several autoimmune and neurodegenerative disorders are in fact associated with ubiquitination proteasome system (UPS) dysregulation and ER dysfunction [118,119,120,121,122]. Furthermore, as an LSD, KD presents defective lysosomes that result in the accumulation of undigested macromolecules, with obvious consequences for proteostasis. In general, failure in removing inherently disordered proteins, such as amyloid-β (Aβ), Tau, and α-synuclein, correlates with distorted protein–protein interactions that trigger event cascades leading to neurological affections [123]. Therefore, the control of protein homeostasis and restoring physiological protein synthesis could represent an attractive therapeutic target for these disorders [124].

ER stress is present from the very first days of life in Twi mice [32] and it may be considered as a hallmark of KD [125]. For this reason, it is extremely interesting to have detected the depletion in Het mice, when compared to both WT and Twi mice, of proteins implicated in the control of ubiquitination and related protein degradation for ER, mitochondrion, and nucleus misfolded proteins, as well as for the maturation of ubiquitin-containing autophagosomes and the clearance of ubiquitinated proteins by autophagy. Subliminal and non-phenotype-affecting proteostasis-dysfunctions, bona fide related to the presence of a mutated *galc* allele, may hence occur in Het young mice, even interesting different PN modules [116]. The decrease in ubiquitin carboxyl-terminal hydrolase 5 (UCHL5) and of the central hub VCP, despite the Het/WT ratio was not significant for the latter, could in fact influence the dynamic regulation and function of the Het neuronal proteome. Over time, this could relate to neurodegenerative disease development, possibly in combination with stressful conditions.

Intriguingly, VCP (Table 1, Figure 2, spot 2) is a lysosomal integrity maintenance protein, retained relevant in LSD [126], and described interrelated with neurological disorders, such as familiar forms of amyotrophic lateral sclerosis (ALS), AD, Parkinson disease (PD), and the inclusion body myopathy with early-onset Paget disease and frontotemporal dementia (IBMPFD) [127]. Among others, it forms a complex with NSFL1 cofactor p47 (p47), another down-regulated protein (Table 1, Figure 2, spot 16) in Het animals, that regulates cell soma ER morphology and function as well as tubular ER formation and extension into dendrites. VCP-p47 defective neurons actually present a reduced number of ER attached ribosomes, decreased protein synthesis, and consequent reduction in dendritic spine density [73].

Moreover, VCP AAA+ ATPase hexamer has “segregase” activity that is required for the endoplasmic reticulum-associated degradation (ERAD) and for releasing specific membrane-bound transcription factors to modulate gene expression, for extracting proteins from the mitochondrial outer membrane and to support mitophagy, and for ribosome, autophagosome, and chromatin-associated protein degradation [95,96,128]. Throughout different cellular compartments, VCP hence exerts a key control on protein synthesis, quality and degradation, also by regulating the clearance of protein aggregates and the autophagy of stress granules [127,129].

Down-stream of VCP and in association with the 19S regulatory particle of the 26S proteasome, UCHL5/UCH37 (Table 1, Figure 2, spot 1) participates in the branched-ubiquitin tag removal from protein substrates, thus controlling their access to the 20S core particle of the proteasome for degradation [130]. This differential ubiquitin tag recognition and removal is highly selective and, as different ubiquitin biopolymer structures differentially prioritize substrates for degradation, vital for proteostasis [131]. In addition, since it exerts its essential role in proteostasis also by editing and rescuing polyubiquitinated proteins before their commitment to the 20S proteasome [121,132,133], UCHL5 modulates cell protein profiles even preventing excessive protein degradation.

**Figure 6:**
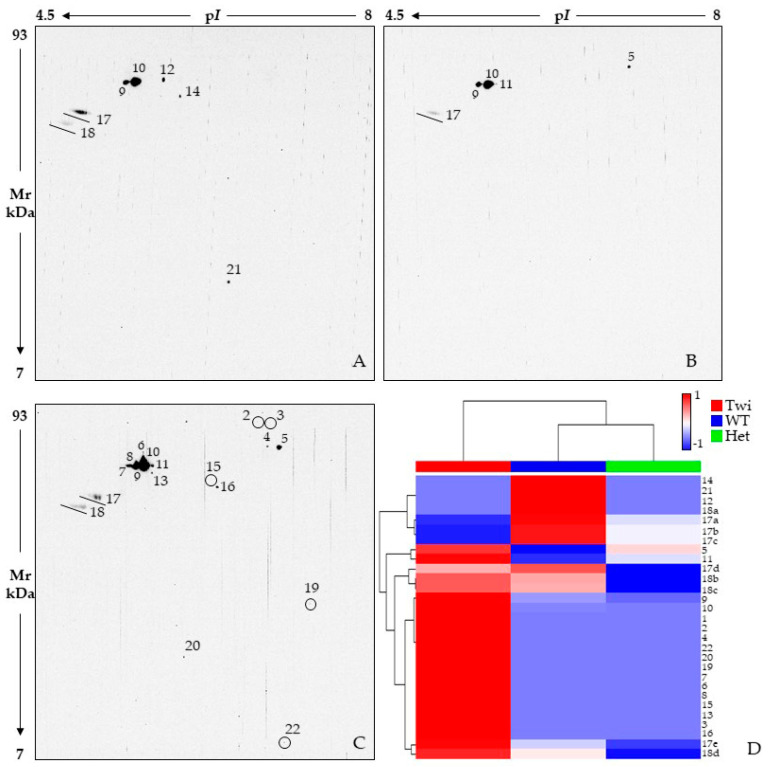
2D Western blot analysis by using anti-ubiquitin antibody on 33-day-old wild-type (WT) (**A**), heterozygous *galc* ^+/−^ (Het) (**B**), and Twitcher (Twi) (**C**) mice. Small signals are circled to facilitate their viewing. Circles are used to improve the visualization of weaker immunoreactive signals (**C**). Heatmap analysis (**D**) performed on scaled values of spot intensity. Color changes from blue to red indicate less or higher signal intensity, respectively. Each row corresponds to a ubiquitinated protein species while each column corresponds to one of the three tested conditions, as highlighted by the colored bar from the horizontal dendrogram: Twi = red, WT = blue, and Het = green. Row spot numbers match those in (**A**–**C**). Spots from the same isoelectric series (i.e., 17 and 18 lines) are named in alphabetical order, from the most acid p*I* value to the most basic one.

UCHL5 activities are finely modulated by its PTMs, which interest several residues of this deubiquitinase (DUB). Their differential occurrence and combination could modify the affinity of UCHL5 with other components of the proteasome as well as with its substrates, thus impacting on its activity and proteasome degradation [123]. Therefore, whether the decrease in the spot 1 proteoform is due to differential modulations of PTMs or due to an overall reduced presence of UCHL5, Het mice most likely have UCHL5 dysfunctions and an ubiquitinated profile affection. In line with this hypothesis, we proved an overall decrease in ubiquitinated proteins in Het mice by performing 2D WB and immunostaining with an anti-ubiquitin primary antibody (Figure 6). Although the ubiquitination pattern of Het mice is quite similar to that of WT, several spots have an evident signal decrease in carries (Figure 6A,B), as highlighted in the immunoreactive-spot intensity heatmap (Figure 6D) and multiple line chart (Supplementary Figure S2). On the contrary, the Twi mouse presents an overall qualitative and quantitative increase in ubiquitinated protein species compared to both WT and Het animals (Figure 6A–C).

The UCHL5 down-regulation observed in KD carriers acquires even more interest given the context of our analyses. UCHL5 actually exerts a crucial role in the NS [134,135]. Not only does the *uchl5* knockout mouse present defects in brain development and is lethal [134], but UCHL5 may act in a neuroprotectant role by modulating NF-κB activation [136,137,138], which is notoriously affected by psychosine [81]. This DUB may also indirectly correlate to amyloid-beta precursor protein (APP) synthesis by deubiquitinating SMADs and type I TGF-β receptor and, consequently, by controlling TGF-β signaling, whose up-regulation associates with AD [139]. In different tissues and cell types, UCHL5 was also described as regulating Wnt/β-catenin signaling and related cellular survival and migration, DNA double-strand break repair, and apoptosis via Bax/Bcl-2, caspase-9, and caspase-3 [131,140]. Interestingly, the Wnt/β-catenin pathway is critical for the NS development [141].

Defects/alterations in UCHL5 activity are hence detrimental to numerous cellular functions and, as a result, the UCHL5 modulation observed in Het mice is unlikely to have no consequences in carriers’ brain physiology.

Besides its role in cytoskeleton organization and signal transduction, RACK1 has also been reported to be active in ubiquitination and proteasome-mediated degradation, as well as in cell cycle arrest and apoptosis induction [142,143].

As mentioned above, NDRG1 is a stress response protein, which widely manipulates the ER stress response and whose expression is induced, among others, by oxidative stress (OS) [102,144]. Twi mice are notoriously under OS [25,145] and the NDRG1 up-regulation we detected in these animals may be, at least in part, due to OS. Since Het animals also present the same abundance increase in the spot 15 proteoform, when compared to the control group, we may speculate that NDRG1 deregulation could similarly impact in Twi and Het mice. Although its function is somewhat controversial, cancer studies reveal that NDRG1 up-regulation reduces the pro-survival response to ER stress by attenuating the PERK-eIF2α pathway of the unfolded protein response (UPR) [102]. The consequent defective autophagy, which is a critical feature in the pathogenesis of KD [146], may further compromise cell brain survival in the Twi mouse.

On the other hand, the NDRG1 increase strengthens the hypothesis of an impaired ability of Het mice to manage stressful and damage-inducing conditions that may cause the formation of hazardous products, e.g., stress granule, whose removal is carried out by autophagy.

### 2.4. Mitochondrial and Energy Supply Dysfunctions

Our comparative proteomics analysis has shown mitochondrial-protein significant affection in brain samples from Twi and Het mice. This improves the delineation in Het animals of mutually-related molecular defects that may result in cellular dysfunctions, relative to several neurodegenerative diseases, strengthening the hypothesis that apparently healthy KD carriers are exposed to an increased risk of developing neuropathies. KD is an LSD caused by a defective lipid and protein metabolism that often presents LB formation [147]. Psychosine induces α-synuclein fibrillization [17] and LB have been recently described as an aberrant protein–lipid compartmentalization presenting a lipid core surrounded by dysmorphic aberrant mitochondria and α-synuclein fibrils [148]. While lipid core is coherent with KD lipid metabolism dyshomeostasis, mitochondrial occurrence in KD LB is in line with other neurological affections presenting LBs and with a consistent body of evidence that proposes mitochondrion dysfunctions playing a relevant rule in this disease onset and development [33,149]. Mitochondrial homeostasis actually depends on the integrity of autophagy and lysosomal degradation that are, as already discussed, broadly affected in KD. At the same time, psychosine has been reported as interfering with mitochondrial functionality in oligodendrocytes by affecting their permeabilization, intra- and extra-mitochondrial Ca^2+^ kinetics, their membrane potential and electron transport chain, oxidative phosphorylation, and cytochrome c release and caspase activation [149,150]. After all, increased ROS production and oxidative stress, largely originated from these defects, are one of the main cytotoxic effects mediated by psychosine. In agreement with the above observations, the MetaCore enrichment analysis that we performed in the “pathway maps” ontology highlighted the significant enrichment of the dihydrolipoyllysine-residue acetyltransferase component of pyruvate dehydrogenase complex (ODP2) and aconitate hydratase (ACO2) protein differences in the “mitochondrial dysfunction in neurodegenerative diseases” pathway.

Mitochondrion aberrances obviously correlate with metabolism dysfunctions and ATP defective production. In particular, mitochondrial ATP production is indispensable for neuronal metabolism and synaptic function: even little defects in ATP synthesis may actually cause excitotoxicity and neuronal death [33]. Pyruvate dehydrogenase complex (PDC) is a crucial enzyme in NS energy metabolism. PDC deficiency has in fact a broad spectrum of clinical features, from severe neonatal/infantile lactic acidosis to chronic neurological disorders, even with later manifestations [151,152]. It links the glycolytic pathway to the Krebs cycle by catalyzing pyruvate conversion to acetyl-CoA and CO_2_. ODP2 is the E2 subunit of the PDC that forms the structural core of the complex. Twi mice have a significant increase in the spot 9 proteoform of ODP2 and a slight increase, despite not being significant, was also detected for Het mice when compared to the controls (Table 1, Figure 2, spot 9). This suggests a deregulation in ODP2 proteoform pattern in Twi animals that, as ODP2 PTMs are vital for the PDC activity [153], may influence the cellular energy supply. The relevance of this enzyme in KD pathogenesis is further stressed by reports describing KD patients with neonatal/infancy and adult onset presenting lactate increases in brain [154,155,156]. Interestingly, glucose hypometabolism and augmented lactate levels were also described in the CNS of patients suffering from other leukodystrophies as well as in symptomatic Twi mice [157]. Noteworthy, MS is also characterized by the low utilization of glucose, most likely a consequence to mitochondrion dysfunction and oxidative stress [158].

Likewise, ACO2 is involved in energy metabolism. It serves as the second step of the tricarboxylic acid (TCA) cycle, where it catalyzes the interconversion of citrate to isocitrate via cis-aconitate. As oxidation of its prosthetic group [4Fe-4S] determines its inactivation, ACO2 is highly vulnerable to ROS attack [159]. Consequently, it is highly likely that ACO2 dysfunctions could occur in Twi mice. Our analysis provided three ACO2 proteoforms (Table 1, Figure 2, spots 3, 4, and 6) whose abundances significantly differ among the three mouse groups. Although differently, ACO2-patterns of both Twi and Het mice diverge from the WT one. This suggests different modulation of its activity even in the carrier *galc ^+/−^* mice. In addition, ACO2 defects could also contribute to mitochondrial dyshomeostasis by interfering in mitochondrion DNA stabilization and mRNA translation [160]. Thus, our results suggest the ACO2 enzyme as a possible biochemical marker of mitochondrial functional impairment in KD and, probably, with a functional relevance, not necessarily negative, also in Het animals.

The ATPB down-regulation detected in Het mice extends the mitochondrial ATP-synthesis alteration to the oxidative phosphorylation pathway. ATPB forms the catalytic core of the structural domain F_1_ of the mitochondrial membrane ATP synthase and catalyzes ATP synthesis. The ATPB proteoform that we identified is consistently down-regulated in Het mice compared to both WT and Twi animals. Although the spot 14 decreased abundance does not imply an overall decrease in this protein, our data suggest, once again, changes in ATPB proteoform pattern that may lead to defective ATP synthesis in heterozygous carriers of *galc* mutations. Of interest, ATPB has been recently detected to be down-regulated in brain samples from PD patients and functionally predicted as one of the main hub gene in the pathogenesis of the disorder [161].

Finally, among the mitochondrial deregulated proteins in mutants, we identified the lipoamide acyltransferase component of branched-chain alpha-keto acid dehydrogenase complex (ODB2) (Table 1, Figure 2, spot 11). It results in being significantly down-regulated in Twi when compared to Het animals and increased, although not significantly, in the latter when compared to the controls. ODB2 is the E2 component of the multi-subunit branched-chain α-ketoacid dehydrogenase (BCKDH), a critical mitochondrial enzyme-complex that provides acetyl-CoA molecules, via oxidative decarboxylation of branched and short-chain alpha-ketoacids, to the TCA cycle. The deregulation of ODB2 not only suggests some alteration in branched-chain amino acid metabolism between Twi and Het animals, but also highlights possible differences in mitochondrial nucleoid dynamics, ODB2 being a core protein of these structures [162]. As a rule, the spot 11 proteoform of ODB2 may be a molecular attempt of Het mice to counteract metabolic aberrances related to the presence of a mutated *galc* allele.

## 3. Materials and Methods

### 3.1. Animal Model

Heterozygous twitcher mice (*galc ^+/−^*) on a congenic C57BLJ/6 background (RRID:IMSR_JAX:000845) were purchased from Jackson Laboratory (Bar Harbor, ME, USA) RRID:IMSR_JAX:000845) [26]. The animals were kept in a controlled environment and conventional conditions at the Department of Molecular and Developmental Medicine, University of Siena. Water and food were continuously available. All mice experiments were conducted according to the protocol approved by the Local Ethical Committee of the University of Siena. All experimental treatments and animal maintenance were carried out according to the Public Health Service (PHS) Policy on Human Care and Use of Laboratory Animals. Homozygous twitcher (*galc ^−/−^*) and homozygous wild-type (*galc ^+/+^*) mice were obtained by crossing heterozygous (*galc ^+/−^*) mice. High Resolution Melting Polymerase Chain Reaction (HRM-PCR) was used to determine newborn mice genotype for the *galc* mutation. Mice were euthanized at the age of 33 days by lethal injection of phenobarbital. Whole brains were collected and, after being thoroughly washed in phosphate buffer saline (PBS), were immediately frozen in liquid nitrogen and stored at −80 °C.

### 3.2. Whole Brain Preparation

Twelve whole brains for each tested condition were collected from 33 day-old Twi, Het, and WT mice. For each condition, samples from seven animals were used for differential 2DE analysis (analytical runs) and those from the other 5 mice were applied in mass spectrometry and western blot analyses. Brain samples were analyzed individually, never pooled. According to [32], proteins were extracted by first pestle-pounding the whole brain in liquid nitrogen and then, by solubilizing resulting dusts in a 2D lysis buffer: 7 M Urea, 2 M Thiourea, 4% (*w*/*v*) 3-[(3-Cholamidopropyl)dimethylammonio]-1-propanesulfonate hydrate (CHAPS), 1% (*w*/*v*) dithioerythritol (DTE) and 0.5% (*w*/*v*) Triton X-100. Obtained lysates were then vortexed and centrifuged at 20,000× *g* for 15 min. Protein concentration was estimated by Bradford assay [163] and aliquots stored at −80°C until use.

### 3.3. Proteomic Analyses

Brain samples were separated by 2DE, according to [32]. For each 2DE analytical run, 60 μg of protein sample with 0.2% (*v*/*v*) of pH 3–10 carrier ampholytes was loaded by cup-loading on isoelectrofocusing (IEF) strips, pH 3–10 non-linear gradient 18 cm in length (Cytiva, formerly GE Healthcare, Marlborough, MA, USA) by the Ettan IPGphor system (Cytiva). For 2DE Western blots (2D WB) and mass spectrometry-compatible 2DE runs, 120 µg and 600 µg, respectively, were loaded with 2% (*v*/*v*) pH 3–10 carrier ampholytes, on the same strip type. Cup loading was performed on IEF strips rehydrated overnight with 350 μL of lysis buffer at room temperature and then IEF was carried out by applying the following voltage settings at 16 °C: 200 V for 8 h; a gradient to 3500 V in 2 h, a step at 3500 V for 2 h, from 3500 V to 5000 V in 2 h, maintained at 5000 V for another 3 h; a gradient to 8000 V in 1 h and a step at 8000 V for 3 h; and finally, a gradient to 10,000 V in 1 h and maintained up to a total of 100,000 V h. After IEF, strips underwent a 12 min equilibration step with a solution composed of 6 M urea, 30% (*v*/*v*) glycerol, 2% (*w*/*v*) sodium dodecyl sulfate (SDS), 0.05 M Tris–HCl pH 6.8 and 2% (*w*/*v*) DTE; and a further 5 min in a solution composed of 6 M urea, 30% (*v*/*v*) glycerol, 2% (*w*/*v*) SDS, 0.05 M Tris–HCl pH 6.8, 2.5% (*w*/*v*) iodoacetamide and trace of bromophenol blue. The second dimension was carried out at 9 °C on 9–16% polyacrylamide linear gradient gels (18 cm × 20 cm × 1.5 mm) at 40 mA/gel. Analytical gels were stained with ammoniacal silver nitrate staining [164], while mass spectrometry preparative gels were stained according to a mass spectrometry-compatible silver staining [165]. Gels images were digitalized using the Image Scanner III by LabScan 6.0 software (GE Healthcare, Chicago, IL, USA) and then analyzed by ImageMaster 2D Platinum v. 6.0 software (Cytiva). The relative volume percentage (%Vol), corresponding to the ratio between the optical density of a single spot (volume) and the total volume of spots present in the same gel, and expressed as a percentage, has been considered for statistical analysis.

### 3.4. Statistical Analysis

Statistical analysis and principal component analysis (PCA) were performed by RStudio Desktop 1.1.463 (Integrated Development for RStudio, Inc., Boston, MA, USA, https://www.rstudio.com (accessed on 1 March 2022)) [166].

Twi, Het, and WT groups were compared using nonparametric statistics. Kruskal–Wallis and Dunn’s Multiple Comparison test with Benjamini–Hochberg post-hoc correction were applied to proteomic datasets. Only spots presenting a *p*-value ≤ 0.05 and a minimal fold change of ±2 in at least one comparison among the three groups were considered as differentially abundant. The %Vol of significantly differing spots were submitted to PCA and the related variance–covariance evaluation was performed on the three first PCs.

### 3.5. Protein Identification by MALDI TOF-TOF Mass Spectrometry

Statistically significant differential spots were manually cut out from mass spectrometry-compatible silver stained gels and destained in a solution of 30 mM potassium ferricyanide and 100 mM sodium sulphate anhydrous and then, in a 200 mM ammonium bicarbonate. Dehydration was carried out in 100% acetonitrile (ACN). Proteins spots were rehydrated and digested in trypsin solution at 37 °C overnight. Obtained peptides were placed on MALDI target, air-dried, and covered with matrix solution of 5 mg/mL α-cyano-4-hydroxycinnamic acid (CHCA) in 50% *(v*/*v)* ACN and 0.5% *(v*/*v)* trifluoroacetic acid (TFA). The mass spectrometry spectra acquisition was carried out utilizing the UltrafleXtreme™ MALDI-TOF/TOF mass spectrometer (Bruker Daltonics, Billerica, MA, USA), equipped with a 200 Hz smartbeam™ I laser (Bruker) in the positive reflector mode with the following parameters: 80 ns of delay; ion source 1: 25 kV; ion source 2: 21.75 kV; lens voltage: 9.50 kV; reflector voltage: 26.30 kV and reflector 2 voltage: 14.00 kV. The applied laser wavelength and frequency were 353 nm and 100 Hz, respectively, and the percentage was set to 50%. Mass spectrometry spectra were processed by the FlexAnalysis software version 3.0 (Bruker), using peptides produced by trypsin autoproteolysis as internal standards for calibration. Common contaminants, such as matrix-related ions, trypsin autolysis and keratin peaks, were removed in the resulting mass lists and protein identification was, then, carried out by Peptide Mass Fingerprinting (PMF) search using MASCOT (Matrix Science Ltd., London, UK, http://www.matrixscience.com (accessed on 20 December 2021)). PMF search was performed setting parameters as follows: *Mus musculus* as taxonomy, SwissProt as database, 50 ppm as mass tolerance, one missed cleavage site, carbamidomethylation of cysteine as fixed modification and oxidation of methionine as a variable modification. Mass spectrometry analyses were performed at the Molsys Technology Platform (http://molsys.dbcf.unisi.it; accessed on 2 May 2022).

### 3.6. Western Blot Analysis

Western blot analysis was performed for proteomic data validations. According to Bianchi et al. [167] after the second dimension run, 2D WB gels were equilibrated in Towbin transfer buffer (25 mM Tris, 192 mM glycine, 20% *(v*/*v)* methanol) and then proteins were electroblotted to nitrocellulose membranes (Hybond ECL, Cytiva). These were thoroughly washed with blotting solution (skimmed milk 3% *(w*/*v)* and Triton X-100 0.1% *(v*/*v)* in PBS pH 7.4) and incubated overnight at 4 °C with the primary antibody. Membranes were tested, individually, with (i) anti-vimentin (D21H3, Abcam, Cambridge, UK) or (ii) anti-ubiquitin (SAB2107745, Sigma, St. Louis, MO, USA), both at 1:1000 dilution. Successively, they were washed with blotting solution and incubated, at room temperature for 2 h, with anti-rabbit IgG (A-4914, Sigma) as secondary antibody, at 1:3000 dilution. Membranes were thoroughly washed: first in the blotting solution, then in Triton X-100 0.5% (*v*/*v*) in PBS pH 7.4, and finally in 0.05 M Tris HCl pH 6.8. Immunoreaction was performed using the ECL chemiluminescence detection system (Cytiva), and signals were detected by exposing membranes to Hyperfilm ECL X-ray films (Cytiva). Images were acquired by the Image Scanner III and the LabScan 6.0 software. Signals were analyzed by using the ImageMaster 2D Platinum v. 6.0 software (Cytiva). The analysis included an automatic detection of immunoreactive signals by filtering from the background and no protein related interfering signals. Density values of detected spots were then exported and analyzed by RStudio Desktop 1.1.463 (Integrated Development for RStudio, Inc., Boston, MA, USA, https://www.rstudio.com).

### 3.7. Enrichment Analysis by MetaCore™ Software

Identified differential proteins were functionally processed by the MetaCore v. 21.3 (Clarivate Analytics, Boston, MA, USA) network building tool using the shortest path algorithm with “high trust interactions” [168,169]. Obtained networks include only closely related proteins: experimental proteins that directly interacted or indirectly mediated only by a non-experimental protein. According to their function, proteins are represented by specific graphic symbols that are reciprocally crosslinked by interaction vectors. We also performed an enrichment analysis of identified differential proteins in the “pathway maps” ontology provided by the MetaCore resource [170].

## 4. Conclusions

This study highlights the heterozygous *galc* ^+/−^ mice showing characteristic proteomic features that clearly distinguish them from Twi and WT mice. Overall, the protein differences we described suggest molecular impairment, probably related to the occurrence of different proteoforms in cytoskeleton dynamics and related signal transduction, the proteostasis network, mitochondrion integrity and functionalities, energy supply, and lipid metabolism.

In particular, the vimentin and RACK1 down-regulation in Het animals may represent a potential molecular link between the KD carrier and the risk of developing neuropathies, principally related to mechanosignaling and intracellular trafficking defects as well as to an altered myelin turnover associated with cytoskeleton dysfunctions. In addition, the dysregulation of NDRG1, besides strengthening our hypothesis on cytoskeletal rearrangement, suggests lipid metabolism defects in Het mice as also proposed for other neurodegenerative disorders, e.g., AD. Furthermore, the overall decrease in ubiquitinated proteins that we detected in Het mice along with the dysregulation of VCP and UCHL5 strengthens the supposition of proteostasis imbalances in KD carriers that may result in a hazard to its NS, classically associated to neuron inclusions and related neurodegenerations. The altered presence of mitochondrial factors, principally ACO2, was also detected in both Twi and Het mice, hence suggesting potential mitochondrion failure with increased oxidative stress and energy imbalance in both Twi and KD carriers.

Nonetheless, the deregulation of some of the identified proteoforms, and especially transgelin-3, MBP and NDRG1 proteoforms, may be also considered a molecular Het mouse response to counterbalance a probable sub-pathological myelin dyshomeostasis in the normal-appearing carriers’ brain, for preserving neuron survival and function. In both cases, the Het proteome profile, by differing from both WT and Twi ones, suggests molecular affections reasonably related to the occurrence of a mutated *galc* allele that could concur over time and, bona fide, in association with stressful conditions to the onset of neurological disorders.

## Figures and Tables

**Table 1:** Significant protein spot differences, from Twi vs. Het, Het vs. WT, and Twi vs. WT comparisons, identified by mass spectrometry.

^a^ Spot N.	^b^ Protein Name	^c^ UniProtKB Accession Number	^d^ MetaCore Name	^e^ Mascot Search Results				^f^ Fold Change		
				Score	Expected	Matched Peptides/ Detected Peptides	Sequence Coverage %	Twi/Het	Het/WT	Twi/WT
1	Ubiquitin carboxyl-terminal hydrolase 5	P56399	USP5	144	6.8 × 10^−11^	14/18	15	4.05 ^¥^	0.22 ^¥^	0.86 ^NS^
2	Transitional endoplasmic reticulum ATPase	Q01853	VCP	226	4.3 × 10^−19^	22/27	25	2.52 ^¥^	0.59 ^NS^	1.48 ^NS^
3	Aconitate hydratase, mitochondrial	Q99KI0	ACON	188	2.7 × 10^−15^	13/14	25	0.70 ^NS^	2.89 ^#^	2.02 ^NS^
4	Aconitate hydratase, mitochondrial	Q99KI0	ACON	100	1.7 × 10^−6^	9/15	16	2.32 ^¥^	1.33 ^NS^	3.07 ^#^
6	Aconitate hydratase, mitochondrial	Q99KI0	ACON	258	2.7 × 10^−22^	20/22	31	2.58 ^¥^	0.72 ^NS^	1.84 ^NS^
7	Albumin	P07724	Albumin	414	6.8 × 10^−38^	29/32	52	2.66 ^NS^	0.26 ^¥^	0.67 ^NS^
9	Dihydrolipoyllysine-residue acetyltransferase component of pyruvate dehydrogenase complex, mitochondrial	Q8BMF4	ODP2	119	2.2 × 10^−8^	10/14	20	1.34 ^NS^	1.50 ^NS^	2 ^¥^
10	Dihydropyrimidinase-related protein 2	O08553	CRMP2	233	8.6 × 10^−20^	17/23	43	2.00 ^#^	0.73 ^NS^	1.45 ^¥^
11	Lipoamide acyltransferase component of branched-chain alpha-keto acid dehydrogenase complex, mitochondrial	P53395	BCKD-E2	127	3.4 × 10^−9^	12/18	17	0.32 ^#^	1.69 ^NS^	0.54 ^NS^
12	2′,3′-cyclic-nucleotide 3′-phosphodiesterase	P16330	CNP1	210	1.7 × 10^−17^	16/25	41	2.47 ^#^	0.47 ^#^	1.14 ^NS^
13	Vimentin	P20152	Vimentin	98	3.00 × 10^−6^	7/9	19	12.75 ^¥^	0.46 ^NS^	5.77 ^¥^
14	ATP synthase subunit beta, mitochondrial	P56480	ATP5B	171	1.4 × 10^−13^	13/17	32	3.98 ^¥^	0.33 ^¥^	1.29 ^NS^
15	Protein NDRG1	Q62433	NDRG1	101	1.4 × 10^−6^	8/9	24	0.91 ^NS^	2.61 ^#^	2.35 ^¥^
16	NSFL1 cofactor p47	Q9CZ44	p47	118	2.7 × 10^−8^	8/12	29	3.74 ^¥^	0.63 ^NS^	2.33 ^¥^
17	Tropomodulin-2	Q9JKK7	Tropomodulin-2	100	1.7 × 10^−6^	9/19	33	3.06 ^¥^	0.28 ^¥^	0.86 ^NS^
18	60S acidic ribosomal protein P0	P14869	RPLP0	194	6.8 × 10^−16^	12/15	47	0.52 ^NS^	2.01 ^¥^	1.03 ^NS^
19	Beta-soluble NSF attachment protein	P28663	NAPB	227	3.4 × 10^−19^	18/22	60	1.67 ^NS^	1.60 ^NS^	2.67 ^¥^
20	Creatine kinase B-type	Q04447	KCRB	127	3.4 × 10^−9^	7/7	21	2.72 ^¥^	0.67 ^NS^	1.82 ^NS^
22	Receptor of activated protein C kinase 1	P68040	RACK1	189	2.2 × 10^−15^	13/23	59	3.14 ^#^	0.42 ^¥^	1.30 ^NS^
23	Protein-L-isoaspartate(D-aspartate) O-methyltransferase	P23506	PIMT	173	8.6 × 10^−14^	12/15	42	0.39 ^¥^	1.71 ^NS^	0.66 ^NS^
24	Translationally-controlled tumor protein	P63028	TPT1	136	4.3 × 10^−10^	10/18	44	1.05 ^NS^	2.7 ^¥^	2.83 ^#^
25	Transgelin-3	Q9R1Q8	Transgelin-3	159	2.2 × 10^−12^	16/30	56	0.53 ^¥^	2.24 ^¥^	1.19 ^NS^
27	Myelin basic protein	P04370	Myelin basic protein	151	1.4 × 10^−11^	10/13	30	0.59 ^¥^	2.12 ^#^	1.25 ^NS^

^a^ Spot numbers match those used in Figure 1 to indicate protein spot differences; ^b^ UniProtKB protein name; ^c^ UniProtKB accession number; ^d^ MetaCore name; ^e^ Mascot search results: number of matched peptides, sequence coverage, and score; ^f^ Fold changes calculated on %Vol values computed for identified spot differences matched in inter-class analysis. Protein differences were considered significant according to a FC ≥ 2 and a *p* ≤ 0.01 (^#^) or 0.01 < *p* ≤ 0.05 (^¥^). NS: not significant.

## Supplementary Materials

The following supporting information can be downloaded at: https://www.mdpi.com/article/10.3390/ijms232113537/s1.

## Abbreviations

%Vol	Percentage relative volume
2DE	Two-dimensional electrophoresis
2D	Two-dimensional
ACN	Acetonitrile
ACO2	Mitochondrial aconitase
AD	Alzheimer disease
ALS	Amyotrophic lateral sclerosis
AP-1	Activator protein 1
APP	Amyloid-beta precursor protein
ATPB	ATP synthase subunit beta, mitochondrial
BMP	Bone morphogenetic protein
CHAPS	3-[(3-cholamidopropyl) dimethylammonio]-1-propanesulfonate
CHCA	α-cyano-4-hydroxycinnamic acid
CNP	2′,3′-cyclic-nucleotide 3′-phosphodiesterase
CNS	Central nervous system
DTE	1,4-Dithioerythritol
DPYSL2	Dihydropyrimidinase-Related Protein 2
DUB	Deubiquitinase
ER	Endoplasmic reticulum
ERAD	Endoplasmic reticulum-associated degradation
FC	Fold change
GALC	Galactosylceramidase
GalCer	Galactosylceramide
GFAP	Glial fibrillary acidic protein
GWAS	Genome-wide association studies
HIF-1A	Hypoxia-inducible factor 1-alpha
Het	Heterozygous mice (*galc ^+/−^*)
HRM-PCR	High resolution melting polymerase chain reaction
IBMPFD	Inclusion body myopathy with early-onset Paget disease and frontotemporal dementia 1
IEF	Isoelectrofocusing
IF	Intermediate filament
KD	Krabbe disease
LacCer	Lactosylceramide
LB	Lewy body
LSD	Lysosomal storage disease
MALDI	Matrix assisted laser desorption/ionization
MAP	Microtubule-associated-protein
MBP	Myelin basic protein
NDRG1	N-myc downstream regulated 1
NS	Nervous system
OS	Oxidative stress
p47	NSFL1 cofactor p47
PBS	Phosphate buffer saline
PC1	First principal component
PC2	Second principal component
PC3	Third principal component
PCA	Principal component analysis
PCs	Principal components
PD	Parkinson disease
PDC	Pyruvate dehydrogenase complex
PHS	Public health service
PKC	Protein kinase C
PFE	Plasma fibrinogen extravasation
PMF	Peptide mass fingerprinting
PN	Proteostasis network
PNS	Peripheral nervous system
PP2A	Protein phosphatase 2A
Psy	Psychosine
PwMS	People with multiple sclerosis
RACK1	Receptor of activated protein c kinase 1
SDS	Sodium dodecyl sulfate
SPN	Shortest-path-network
STAT1	Activator of transcription-1
STAT3	Activator of transcription-3
TCA	Tricarboxylic acid
TCTP	Translationally-controlled tumor protein
TOF	Time of flight
TW	Homozygous twitcher mice (*galc ^−/−^)*
UCHL5	Ubiquitin carboxyl-terminal hydrolase 5
UPS	Ubiquitination proteasome system
VCP	Transitional endoplasmic reticulum ATPase; alternative name: valosin-containing protein
VIME	Vimentin
WB	Western blot
WT	Homozygous wild-type mice (*galc ^+/+^*)
