# Supplementary material for: Neurodegenerative Disorder Risk in Krabbe Disease Carriers
Source: Int J Mol Sci. 2022 Nov 4;23(21):13537. doi: 10.3390/ijms232113537 (PMC9654610; doi:10.3390/ijms232113537)
Supplement: Supplementary file 1 [file ijms-23-13537-s001.zip › Table S1.pdf]

Table S1. Enrichment by Pathway Maps. MetaCore Analysis: canonical pathways by gene ontology of the differential proteins among the comparisons

#	Maps	Total	pValue	Min FDR	p-value	FDR	*In* Data	Network Objects
1	Development_Neural stem cell lineage commitment (schema)	38	1,045E-03	8,609E-02	1,045E-03	8,609E-02	2	Vimentin, Myelin basic protein
2	Mitochondrial dysfunction in neurodegenerative diseases	59	2,505E-03	8,609E-02	2,505E-03	8,609E-02	2	ODP2, ACON
3	Transcription_Negative regulation of HIF1A function	69	3,409E-03	8,609E-02	3,409E-03	8,609E-02	2	VCP, RACK1
4	Development_Positive regulation of WNT/Beta-catenin signaling in the nucleus	69	3,409E-03	8,609E-02	3,409E-03	8,609E-02	2	USP5, VCP
5	Main pathways of Schwann cells transformation in neurofibromatosis type 1	80	4,555E-03	9,197E-02	4,555E-03	9,197E-02	2	Myelin basic protein, TPT1
6	Transport_RAB1A regulation pathway	12	1,522E-02	9,197E-02	1,522E-02	9,197E-02	1	SNAPs
7	SHH signaling in oligodendrocyte precursor cells differentiation in multiple sclerosis	13	1,647E-02	9,197E-02	1,647E-02	9,197E-02	1	Myelin basic protein
8	Development_Schema: Adult neurogenesis in the Subventricular Zone	19	2,399E-02	9,197E-02	2,399E-02	9,197E-02	1	Vimentin
9	Cell adhesion_Desmosomes	19	2,399E-02	9,197E-02	2,399E-02	9,197E-02	1	Vimentin
10	Regulation of degradation of wtCFTR	20	2,524E-02	9,197E-02	2,524E-02	9,197E-02	1	VCP
11	Retinoic acid regulation of oligodendrocyte differentiation in multiple sclerosis	24	3,022E-02	9,197E-02	3,022E-02	9,197E-02	1	Myelin basic protein
12	Zinc deficiency in Prostate Cancer	25	3,146E-02	9,197E-02	3,146E-02	9,197E-02	1	ACON
13	Cell adhesion_Endothelial cell contacts by junctional mechanisms	26	3,270E-02	9,197E-02	3,270E-02	9,197E-02	1	Vimentin
14	Pancreatic cancer cell resistance to Tarceva (erlotinib)	27	3,393E-02	9,197E-02	3,393E-02	9,197E-02	1	Vimentin
15	Translation_Regulation of translation initiation	27	3,393E-02	9,197E-02	3,393E-02	9,197E-02	1	RACK1
16	Rho-dependent regulation of normal and asthmatic smooth muscle contraction	28	3,517E-02	9,197E-02	3,517E-02	9,197E-02	1	RACK1
17	Development_Astrocyte differentiation (general schema)	28	3,517E-02	9,197E-02	3,517E-02	9,197E-02	1	Vimentin
18	Notch signaling in oligodendrocyte precursor cell differentiation in multiple sclerosis	29	3,640E-02	9,197E-02	3,640E-02	9,197E-02	1	Myelin basic protein
19	Development_Transcription factors in segregation of hepatocytic lineage	30	3,764E-02	9,197E-02	3,764E-02	9,197E-02	1	Albumin
20	Mechanisms of K-RAS addiction in lung cancer cells	31	3,887E-02	9,197E-02	3,887E-02	9,197E-02	1	Vimentin
21	SHH signaling in colorectal cancer	31	3,887E-02	9,197E-02	3,887E-02	9,197E-02	1	Vimentin
22	Role of inhibition of WNT signaling in the progression of lung cancer	31	3,887E-02	9,197E-02	3,887E-02	9,197E-02	1	Vimentin
23	Development_FGF and BMP signaling in early embryonic hepatogenesis	31	3,887E-02	9,197E-02	3,887E-02	9,197E-02	1	Albumin
24	WNT signaling in invasive-type melanoma cells	32	4,010E-02	9,197E-02	4,010E-02	9,197E-02	1	Vimentin
25	Inhibition of LKB1 / AMPK signaling in breast cancer	32	4,010E-02	9,197E-02	4,010E-02	9,197E-02	1	Vimentin
26	Interaction of deficient alpha-MSH signaling with TNF-alpha in melanoma	33	4,132E-02	9,197E-02	4,132E-02	9,197E-02	1	Vimentin
27	Prolactin signaling in Prostate Cancer	33	4,132E-02	9,197E-02	4,132E-02	9,197E-02	1	ACON
28	Development_Self-renewal and pluripotency maintenance of human embryonic stem cells	33	4,132E-02	9,197E-02	4,132E-02	9,197E-02	1	Albumin
29	SHH signaling in melanoma	33	4,132E-02	9,197E-02	4,132E-02	9,197E-02	1	Vimentin
30	Development_FGF2 signaling during embryonic stem cell differentiation	34	4,255E-02	9,197E-02	4,255E-02	9,197E-02	1	Albumin
31	Development_Role of cell-cell and ECM-cell interactions in oligodendrocyte differentiation and myelination	34	4,255E-02	9,197E-02	4,255E-02	9,197E-02	1	Myelin basic protein
32	Development_Epigenetic and transcriptional regulation of oligodendrocyte precursor cell differentiation and myelination	34	4,255E-02	9,197E-02	4,255E-02	9,197E-02	1	Myelin basic protein
33	Development_TGF-beta-dependent induction of EMT via SMADs	35	4,378E-02	9,197E-02	4,378E-02	9,197E-02	1	Vimentin
34	Development_Schema: Adult neuron differentiation in the Subventricular and Subgranular Zones	35	4,378E-02	9,197E-02	4,378E-02	9,197E-02	1	Vimentin

35	Cytoskeleton remodeling_Keratin filaments	36	4,500E-02	9,197E-02	4,500E-02	9,197E-02	1	Vimentin
36	Propionate metabolism p.1	36	4,500E-02	9,197E-02	4,500E-02	9,197E-02	1	BCKD-E2
37	Development_Retinoic acid and retinoic acid receptors in regulation of oligodendrocyte differentiation	37	4,622E-02	9,197E-02	4,622E-02	9,197E-02	1	Myelin basic protein
38	Immune response_Role of integrins in NK cells cytotoxicity	38	4,744E-02	9,197E-02	4,744E-02	9,197E-02	1	RACK1
39	Development_Oligodendrocyte differentiation (general schema)	38	4,744E-02	9,197E-02	4,744E-02	9,197E-02	1	Myelin basic protein
40	Regulation of degradation of deltaF508-CFTR in CF	39	4,866E-02	9,197E-02	4,866E-02	9,197E-02	1	VCP
41	Recurrent gene fusions in Prostate Cancer	40	4,988E-02	9,197E-02	4,988E-02	9,197E-02	1	NDRG1
42	Development_Astrocyte differentiation from adult stem cells	40	4,988E-02	9,197E-02	4,988E-02	9,197E-02	1	Vimentin
43	Signal transduction_Role of MIF as an intracellular mediator	41	5,110E-02	9,197E-02	5,110E-02	9,197E-02	1	Vimentin
44	Stromal-epithelial interaction in Prostate Cancer	42	5,231E-02	9,197E-02	5,231E-02	9,197E-02	1	Vimentin
45	Development_FGF signaling in pancreatic and hepatic differentiation of embryonic stem cells	42	5,231E-02	9,197E-02	5,231E-02	9,197E-02	1	Albumin
46	Immune response_Antimicrobial actions of IFN-gamma	43	5,353E-02	9,197E-02	5,353E-02	9,197E-02	1	ACON
47	Leucine, isoleucine and valine metabolism	43	5,353E-02	9,197E-02	5,353E-02	9,197E-02	1	BCKD-E2
48	Transition of HCC cells to invasive and migratory phenotype	44	5,474E-02	9,197E-02	5,474E-02	9,197E-02	1	Vimentin
49	TGF-beta 1-mediated induction of EMT in normal and asthmatic airway epithelium	44	5,474E-02	9,197E-02	5,474E-02	9,197E-02	1	Vimentin
50	Inhibition of remyelination in multiple sclerosis: regulation of cytoskeleton proteins	44	5,474E-02	9,197E-02	5,474E-02	9,197E-02	1	Myelin basic protein
