# Supplementary figures and images for: Neurodegenerative Disorder Risk in Krabbe Disease Carriers
Source: Int J Mol Sci. 2022 Nov 4;23(21):13537. doi: 10.3390/ijms232113537 (PMC9654610; doi:10.3390/ijms232113537)

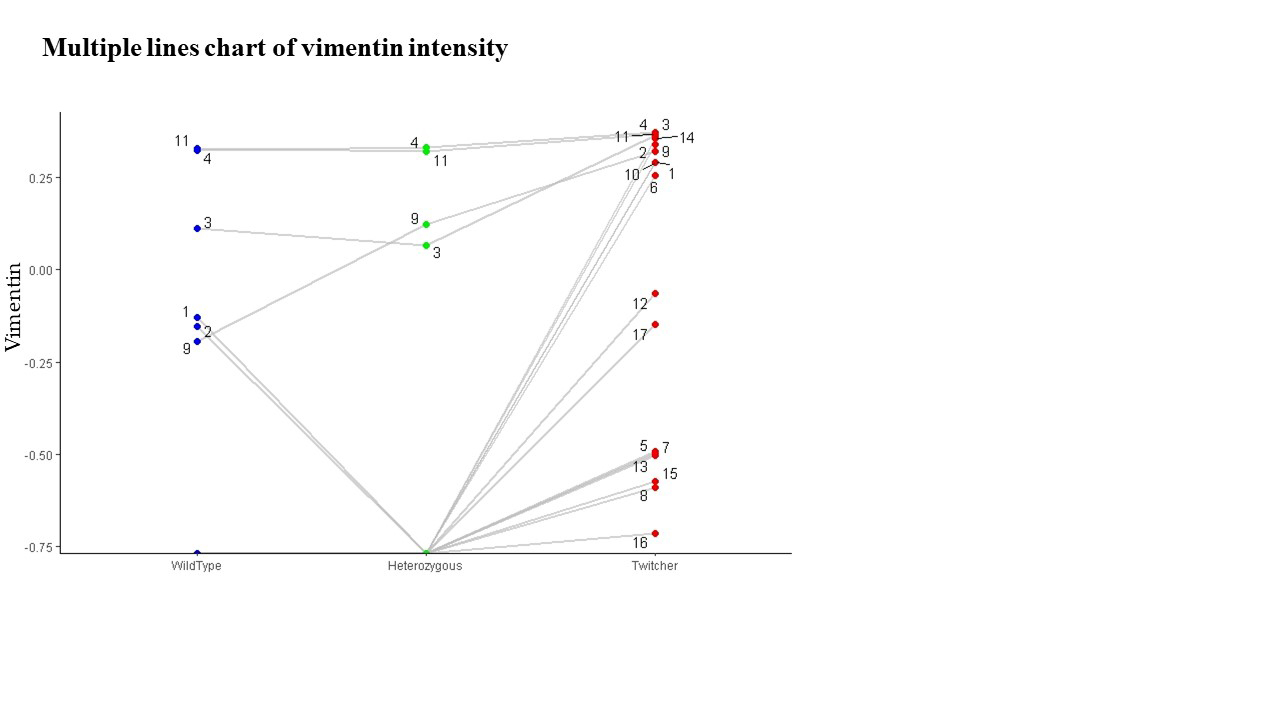

Supplement: Supplementary file 1 [file ijms-23-13537-s001.zip › Figure S1.jpg]

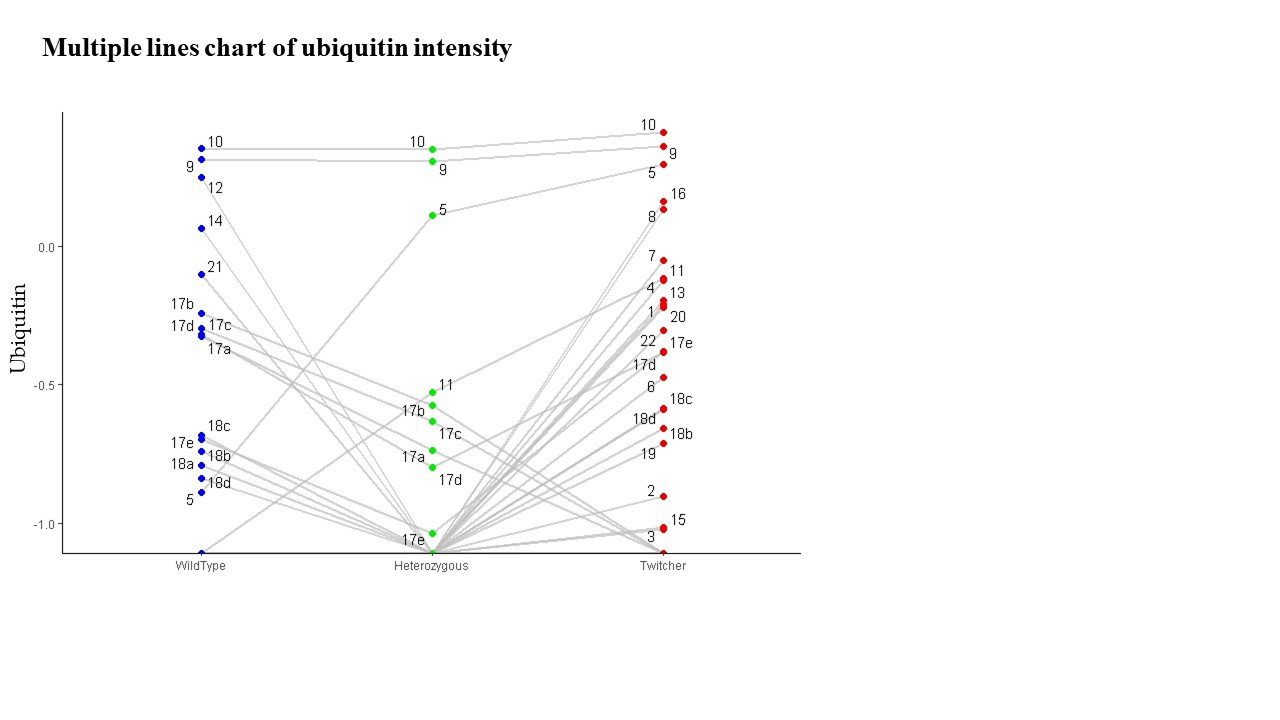

Supplement: Supplementary file 1 [file ijms-23-13537-s001.zip › Figure S2.jpg]
